# Supplementary figures and images for: A novel genotype of Hantaan orthohantavirus harbored by Apodemus agrarius chejuensis as a potential etiologic agent of hemorrhagic fever with renal syndrome in Republic of Korea
Source: PLoS Negl Trop Dis. 2021 May 12;15(5):e0009400. doi: 10.1371/journal.pntd.0009400 (PMC8143423; doi:10.1371/journal.pntd.0009400)

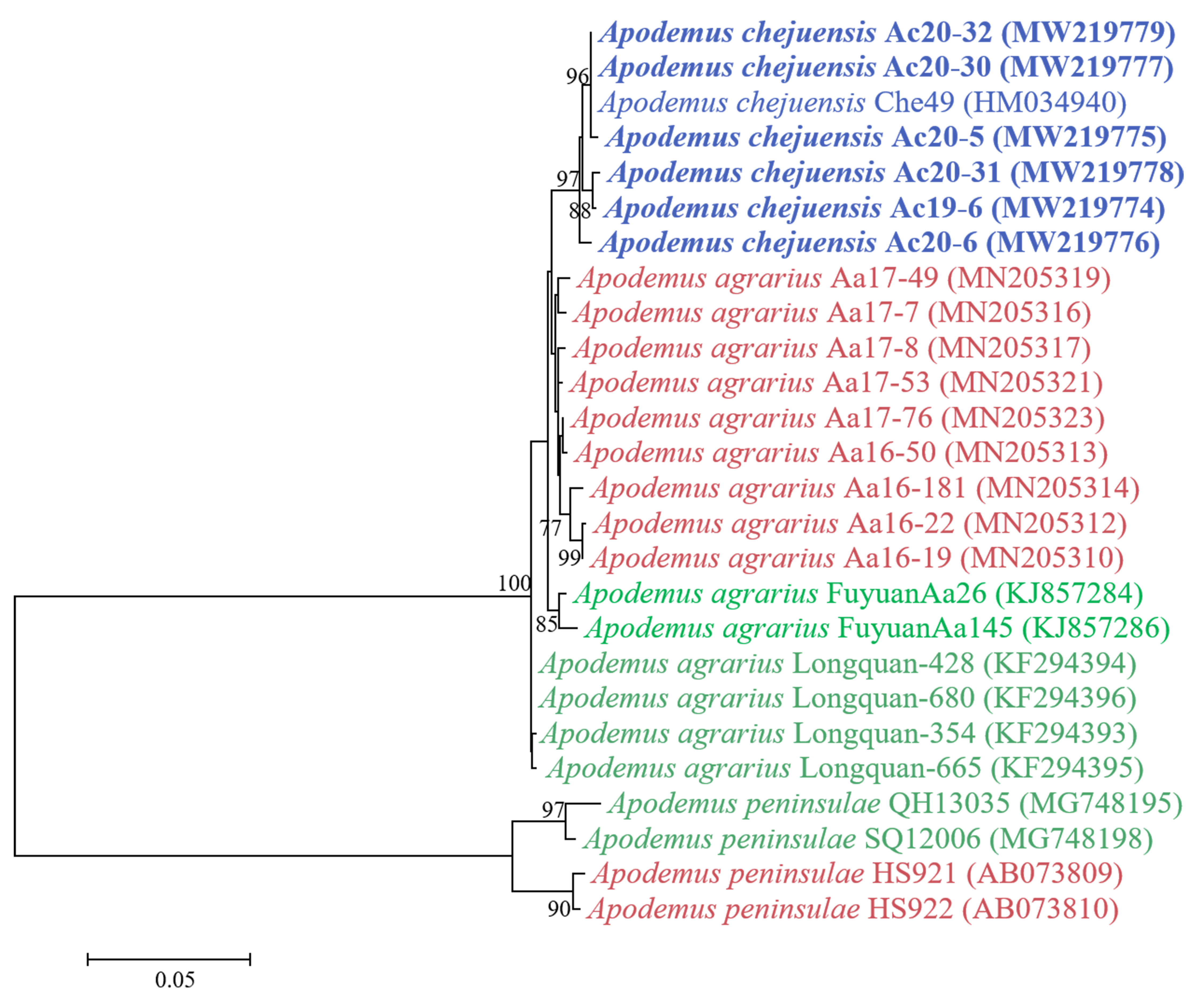

Supplement: S1 Fig — Jeju striped field mice (A. chejuensis) from Jeju Island were confirmed by conventional polymerase chain reaction (PCR) for the mitochondrial DNA cytochrome b gene (coordinates 119–1,080 nt). The phylogenetic tree was generated by the maximum likelihood method using MEGA 7.0. A bootstrap support value >70% is described at the nodes. Apodemus mice are color-coded according to geographic regions (red, mainland ROK; blue, Jeju Island of ROK; green, China). (TIF) [file pntd.0009400.s001.tif]

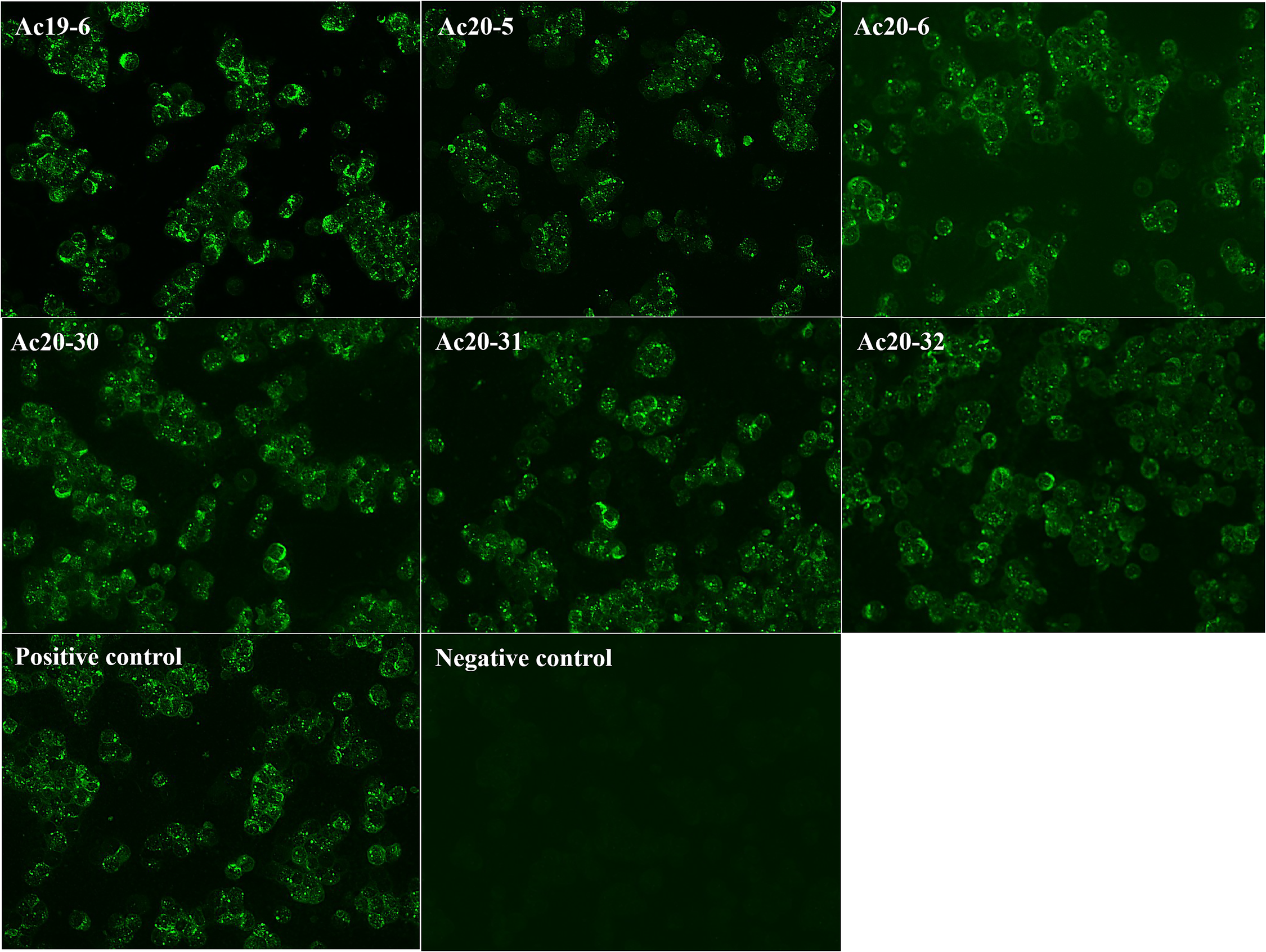

Supplement: S2 Fig — HTNV-infected Vero E6 cells were fixed on each slide. The sera (1:32 dilution) or heart fluids (1:2 dilution) of rodents were used to detect anti-HTNV IgG by IFA, Ac18-6, Ac18-17, Ac19-6, Ac20-30, and Ac20-31; heart fluids of Ac20-5 and Ac20-6. Positive and negative controls used were serum (Aa18-185) and phosphate buffered saline (PBS), respectively. Ac, A. chejuensis; Aa, A. agrarius. (TIF) [file pntd.0009400.s002.tif]

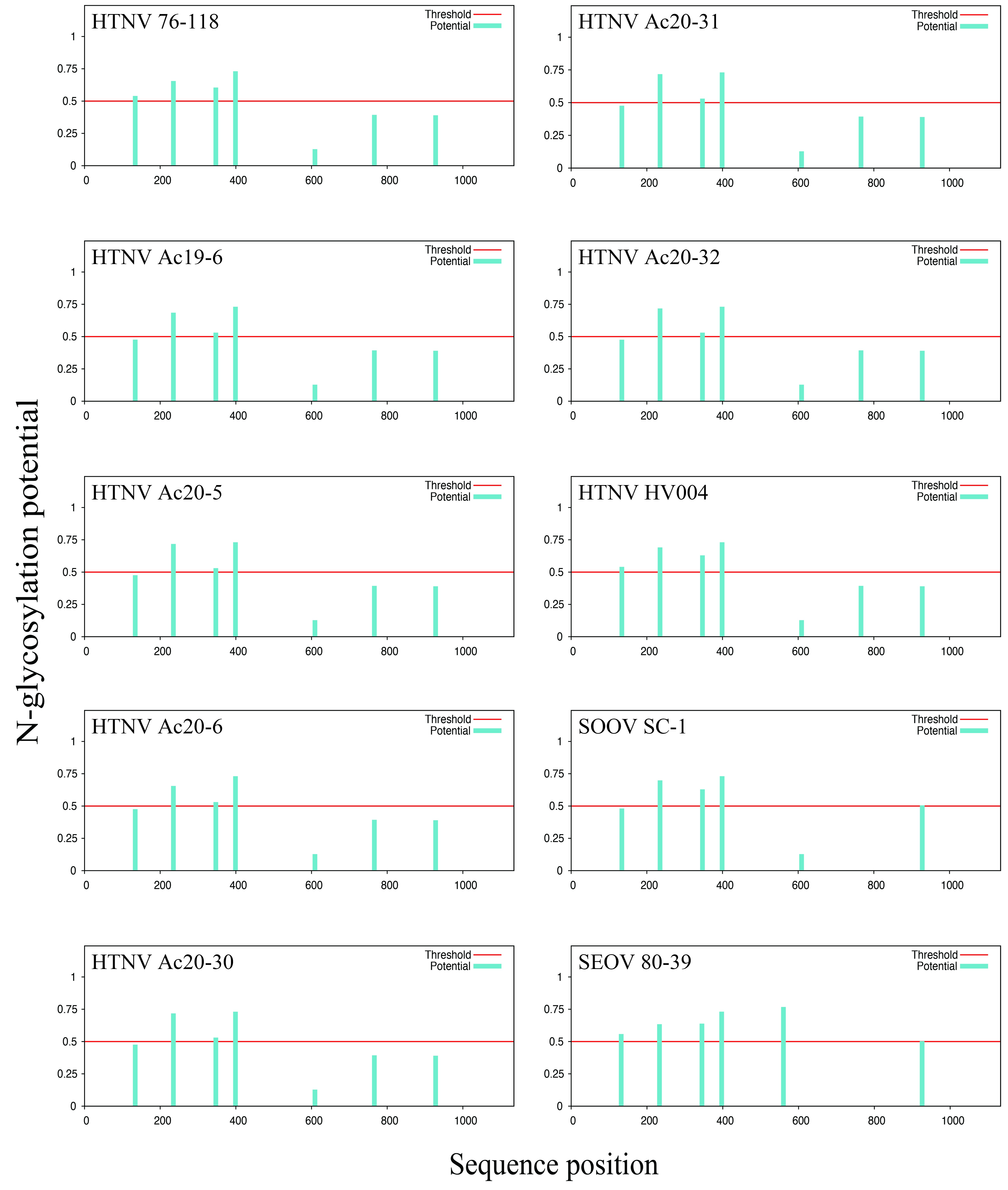

Supplement: S3 Fig — The N-linked glycosylation sites for the entire length of glycoproteins in hantaviruses were predicted using NetNGlyc 1.0 server (DTU Bioinformatics), and the parameter was set to default. The potential N-linked glycosylation sites for rodent-borne orthohantaviruses (HTNV, SOOV, and SEOV) are shown. HTNV, Hantaan virus; SOOV, Soochong virus; SEOV, Seoul virus. (TIF) [file pntd.0009400.s003.tif]
