# Supplementary material for: A novel genotype of Hantaan orthohantavirus harbored by Apodemus agrarius chejuensis as a potential etiologic agent of hemorrhagic fever with renal syndrome in Republic of Korea
Source: PLoS Negl Trop Dis. 2021 May 12;15(5):e0009400. doi: 10.1371/journal.pntd.0009400 (PMC8143423; doi:10.1371/journal.pntd.0009400)
Supplement: S1 Table — (PDF) [file pntd.0009400.s004.pdf]

S1 Table. Summary of mapped reads and depth of coverages by Hantaan virus (HTNV) multiplex PCR-based next-generation sequencing.

Sample	Total reads	Mapped reads / Total reads (%)	L segment		M segment		S segment	
			Mapped reads (%)	Depth of coverage	Mapped reads (%)	Depth of coverage	Mapped reads (%)	Depth of coverage
Ac19-6	720,838	11.8	2,794 (0.4)	64.6	35,966 (5.0)	1,501.9	46,629 (6.5)	4,151.5
Ac20-5	2,287,084	56.3	21,516 (0.9)	497.3	755,097 (33.0)	31,532.0	511,299 (22.4)	45,522.5
Ac20-6	1,597,734	28.5	6,276 (0.4)	145.1	27,269 (17.1)	1,138.7	421,325 (26.4)	37,511.8
Ac20-30	1,141,262	44.6	30,952 (2.7)	715.4	297,141 (26.0)	12,408.3	180,933 (15.9)	16,109.0
Ac20-31	1,547,806	32.5	32,313 (2.1)	746.9	359,727 (23.2)	15,021.8	111,552 (7.2)	9,931.8
Ac20-32	2,615,908	30.5	53,036 (2.0)	1,225.8	549,311 (21.0)	22,938.6	195,606 (7.5)	17,415.4
**Average**	**1,651,772**	**34.0**	**24,481 (1.5)**	**565.9**	**337,419 (20.4)**	**16,607.9**	**244,557 (14.8)**	**21,773.7**

Ac, *Apodemus chejuensis*; C_t, cycle threshold; L, large; M, medium; S, small.

The depth of coverage was determined by the number of mapped reads (read length × number of reads matching the reference/reference genome size).
