# Supplementary material for: A novel genotype of Hantaan orthohantavirus harbored by Apodemus agrarius chejuensis as a potential etiologic agent of hemorrhagic fever with renal syndrome in Republic of Korea
Source: PLoS Negl Trop Dis. 2021 May 12;15(5):e0009400. doi: 10.1371/journal.pntd.0009400 (PMC8143423; doi:10.1371/journal.pntd.0009400)
Supplement: S2 Table — (PDF) [file pntd.0009400.s005.pdf]

S2 Table. Percentage similarity based on L segment of HTNV nucleotide and amino acid sequences between HTNV from Jeju Island and representative rodent-borne orthohantaviruses.

	Homology (%)							
**Sample (Host)**	**HTNV 76-118 (*A. agrarius*)**		**HTNV HV004 (*A. agrarius*)**		**SOOV SC1 (*A. peninsulae*)**		**SEOV 80-39 (*R. norvegicus*)**	
	**nt (%)**	**aa (%)**	**nt (%)**	**aa (%)**	**nt (%)**	**aa (%)**	**nt (%)**	**aa (%)**
Ac19-6	82.6	96.1	81.4	95.4	81.0	95.2	74.5	83.8
Ac20-5	82.4	96.8	81.6	96.1	81.3	95.9	74.6	84.6
Ac20-6	82.2	97.1	81.6	96.2	81.3	96.0	74.7	84.8
Ac20-30	82.9	97.2	82.0	96.4	81.6	96.2	74.8	84.8
Ac20-31	83.1	97.0	82.1	96.2	81.4	95.8	74.7	84.6
Ac20-32	82.9	97.0	81.8	96.0	81.1	95.8	74.8	84.8

HTNV, Hantaan virus; SOOV, Soochong virus; SEOV, Seoul virus; nt, nucleotide; aa, amino acid.
