# Supplementary material for: A novel genotype of Hantaan orthohantavirus harbored by Apodemus agrarius chejuensis as a potential etiologic agent of hemorrhagic fever with renal syndrome in Republic of Korea
Source: PLoS Negl Trop Dis. 2021 May 12;15(5):e0009400. doi: 10.1371/journal.pntd.0009400 (PMC8143423; doi:10.1371/journal.pntd.0009400)
Supplement: S3 Table — (PDF) [file pntd.0009400.s006.pdf]

S3 Table. Percentage similarity based on M segment of HTNV nucleotide and amino acid sequences between HTNV from Jeju Island and representative rodent-borne orthohantaviruses.

	Homology (%)							
**Sample (Host)**	**HTNV 76-118 (*A. agrarius*)**		**HTNV HV004 (*A. agrarius*)**		**SOOV SC1 (*A. peninsulae*)**		**SEOV 80-39 (*R. norvegicus*)**	
	**nt (%)**	**aa (%)**	**nt (%)**	**aa (%)**	**nt (%)**	**aa (%)**	**nt (%)**	**aa (%)**
Ac19-6	88.3	97.6	84.0	96.7	80.1	90.9	70.8	76.9
Ac20-5	87.2	97.5	83.8	96.8	80.3	91.2	70.9	76.6
Ac20-6	88.8	98.2	83.9	97.1	80.4	91.5	70.9	77.0
Ac20-30	87.3	97.7	83.9	97.0	80.3	91.5	71.0	76.9
Ac20-31	87.3	97.6	83.9	97.0	80.3	91.5	71.0	76.8
Ac20-32	87.4	97.7	83.9	97.0	80.3	91.5	71.0	76.9

Ac, *Apodemus chejuensis;* HTNV, Hantaan virus; SOOV, Soochong virus; SEOV, Seoul virus; nt, nucleotide; aa, amino acid.
