# Supplementary material for: A novel genotype of Hantaan orthohantavirus harbored by Apodemus agrarius chejuensis as a potential etiologic agent of hemorrhagic fever with renal syndrome in Republic of Korea
Source: PLoS Negl Trop Dis. 2021 May 12;15(5):e0009400. doi: 10.1371/journal.pntd.0009400 (PMC8143423; doi:10.1371/journal.pntd.0009400)
Supplement: S4 Table — (PDF) [file pntd.0009400.s007.pdf]

S4 Table. Percentage similarity based on S segment of HTNV nucleotide and amino acid sequences between HTNV from Jeju Island and representative rodent-borne orthohantaviruses.

	Homology (%)							
**Sample (Host)**	**HTNV 76-118 (*A. agrarius*)**		**HTNV HV004 (*A. agrarius*)**		**SOOV SC1 (*A. peninsulae*)**		**SEOV 80-39 (*R. norvegicus*)**	
	**nt (%)**	**aa (%)**	**nt (%)**	**aa (%)**	**nt (%)**	**aa (%)**	**nt (%)**	**aa (%)**
Ac19-6	85.4	96.3	85.3	97.2	84.3	96.3	69.6	82.3
Ac20-5	84.8	96.5	85.0	97.4	84.5	96.5	69.7	82.5
Ac20-6	84.9	96.5	85.1	97.4	84.6	96.5	69.7	82.5
Ac20-30	85.4	96.3	85.3	97.2	84.6	96.3	69.6	82.3
Ac20-31	85.1	96.5	85.1	97.0	84.8	96.0	69.8	82.5
Ac20-32	85.1	96.7	85.1	97.2	84.5	96.3	69.6	82.8

Ac, *Apodemus chejuensis;* HTNV, Hantaan virus; SOOV, Soochong virus; SEOV, Seoul virus; nt, nucleotide; aa, amino acid.
