# Supplementary material for: A novel genotype of Hantaan orthohantavirus harbored by Apodemus agrarius chejuensis as a potential etiologic agent of hemorrhagic fever with renal syndrome in Republic of Korea
Source: PLoS Negl Trop Dis. 2021 May 12;15(5):e0009400. doi: 10.1371/journal.pntd.0009400 (PMC8143423; doi:10.1371/journal.pntd.0009400)
Supplement: S5 Table — (PDF) [file pntd.0009400.s008.pdf]

S5 Table. Topography and GPS coordinates of the trapping sites.

Year	Trapping site	Topography	GPS coordinates
2018	Ara-dong	herbaceous vegetations, Hill, and mountains	33°27′55″ N 126°32′45″ E
	Aewol-eup	herbaceous vegetations and farmlands	33°26′6″ N 126°20′32″ E
	Ora-dong	unmanaged grasses, mountains, and forests	33°27′12″ N 126°30′38″ E
2019	Haengwon-ri	unmanaged grasses, farmlands	33°33′3″ N 126°48′41″ E
	Ora-dong	unmanaged grasses, mountains, and forests	33°27′11″ N 126°30′38″ E
2020	Bongseong-ri	herbaceous vegetations and farmlands	33°24′51″ N 126°18′19″ E
	Haengwon-ri	unmanaged grasses, farmlands	33°33′4″ N 126°48′41″ E
	Ora-dong	Field and forest	33°27′09·31″ N 126°30′39·10″ E
	Yongsu-ri	unmanaged grasses, farmlands	33°19′3″ N 126°10′50″ E
