# Supplementary material for: A novel genotype of Hantaan orthohantavirus harbored by Apodemus agrarius chejuensis as a potential etiologic agent of hemorrhagic fever with renal syndrome in Republic of Korea
Source: PLoS Negl Trop Dis. 2021 May 12;15(5):e0009400. doi: 10.1371/journal.pntd.0009400 (PMC8143423; doi:10.1371/journal.pntd.0009400)
Supplement: S6 Table — (PDF) [file pntd.0009400.s009.pdf]

S6 Table. Oligonucleotide primer sequences generated in this study.

Target segment	Primer name	Primer sequence (5′ → 3′)	Product size (bp)	Reference
L segment	HAN-L-F1[a]	5′-ATG TAY GTB AGT GCW GAT GC-3′	452	
	HAN-L-R1[a]	5′-AAC CAD TCW GTY CCR TCA TC-3′		
	HAN-L-F2[a]	5′-TGC WGA TGC HAC IAA RTG GTC-3′	389	
	HAN-L-R2[a]	5′-GCR TCR TCW GAR TGR TGD GCA A-3′		
M segment	JJ-HTNV-M-F-outer[a]	5′-TGG AAT GAC AAT GCT CAT GGA GT-3′	343	
	JJ-HTNV-M-R-outer[a]	5′-GGA CAA TCT GAT GGA TTA CAA CCC C-3′		
	JJ-HTNV-M-F-inner[a]	5′-CCT ATG CAC ACA GAT TTG GAA CT-3′	262	
	JJ-HTNV-M-R-inner[a]	5′-GTT CAT AGT GGC ATT TTG CAG TAT GC-3′		
S segment	HFRS-S-2F[a]	5′-ARA RRT CAR BVC THA GBT AYG-3′	1,009	[1]
	HFRS-S-2R[a]	5′-TGR TTV GAK ATT TCC TTS AC-3′		
	HFRS-S2nd-1F[a]	5′-GAY ATT GAW GAA CCW ASW GGV C-3′	725	
	HFRS-S2nd-1R[a]	5′-GAH GCC ATK AT GTR TTY CKC-3′		
S segment	JJ_HTN_S-229F[b]	5′- CTG GCA GAT AGG ATT GCA AC -3′	153	
	JJ_HTN_S-381R[b]	5′- CTG TCC AGT AGG TTC ATC AAT ATC-3′		

[a]: Oligonucleotide primers for reverse transcription-polymerase chain reaction (RT-PCR)

[b]: Oligonucleotide primers for real-time quantitative transcription-polymerase chain reaction (RT-qPCR)

1. Kim D, Jalal S, Kim C, Song H, Lee J, Shin M, et al. Geographical clustering of hantavirus isolates from *Apodemus agrarius* identified in the Republic of Korea indicate the emergence of a new hantavirus genotype. [Preprint]. 2020. Available from: https://www.researchsquare.com/article/rs-12650/v1.
