# Supplementary material for: A novel genotype of Hantaan orthohantavirus harbored by Apodemus agrarius chejuensis as a potential etiologic agent of hemorrhagic fever with renal syndrome in Republic of Korea
Source: PLoS Negl Trop Dis. 2021 May 12;15(5):e0009400. doi: 10.1371/journal.pntd.0009400 (PMC8143423; doi:10.1371/journal.pntd.0009400)
Supplement: Attachment — Submitted filename: PNTD_HFRS_REVIEW.docx [file pntd.0009400.s010.docx]

**20-01-2021**

The study entitled “A novel genotype of *Apodemus chejuensis*-borne Hantaan Orthohantavirus as a potential etiologic agent of Hemorrhagic Fever with Renal Syndrome in Republic of Korea” appears to be a fascinating approach to identify the novel genotype as a pivotal etiologic agent of HFRS.

However, there are certain minor areas in which the author needs to clarify in order to refine the work for better representation.

1. Line no. 25 – Repetition of the word “pulmonary”. Hantavirus cardiopulmonary syndrome (HCP)
2. Line 34 - The expression of sentence is not very clear. What does the author mean to say, should clearly explain?
3. Line 87 - HFRS-Borne or Rodent-borne? HTNV is the causative agent of HFRS.

- What does the author mean to say by the phrase clinical HFRS-borne HTNV strain?

4. Line 95 – 64 should be replaced with sixty-four.
5. Line 108 - The author has mentioned about the collection of shrews and rodents both for the experiment. But the data related to shrews are missing.

- Was there any seropositive case for shrews as well?

6. Line 124 – What was the estimated cut-off value for the RT-qPCR. Author did not mention about that.
