# Supplementary material for: A novel genotype of Hantaan orthohantavirus harbored by Apodemus agrarius chejuensis as a potential etiologic agent of hemorrhagic fever with renal syndrome in Republic of Korea
Source: PLoS Negl Trop Dis. 2021 May 12;15(5):e0009400. doi: 10.1371/journal.pntd.0009400 (PMC8143423; doi:10.1371/journal.pntd.0009400)
Supplement: Attachment — Submitted filename: 4.PNTD-D-20-02190_Minor Revision_20210409.docx [file pntd.0009400.s011.docx]

**Point by point**

**Minor revision**

First, we want to thank the reviewers for the helpful comments on our original submission. We have responded to all the suggestions and commentaries and this has certainly resulted in an improved manuscript. We have marked with highlighted and burgundy color font those sections of the manuscript that directly answer the reviewers concerns. Below is a detailed point by point response to the reviewer’s comments.

**1.** Abstract, line 37: "The HTNV genomic sequences were phylogenetically related to a viral sequence from the patients with HFRS in southern ROK" - wouldn't this be more precise to say "patient" instead of "patients" since there was only one sequence available?

**R**: Thank you for your point. Based on your suggestion, the term was corrected to patient from patients. [Line 38].

**2.** Abstract, lines 89-90: "We also highlight preemptive precautions for preventing and controlling hantaviral outbreaks to physicians on Jeju Island" I don't recall seeing much in the way of preemptive precautions in the manuscript - this may need to be drawn out a bit more in a couple of sentences in the Discussion.

**R**: Thank you for a good comment. We modified the sentence as shown below [Line 90-92].

**Line 90-92:**

We highlight the necessity of continued surveillance and large-scale investigations for understanding the evolutionary diversification, transmission, and pathogenicity of orthohantaviruses in the southern areas, ROK.

**3.** Results, lines 121-122: "Both IFA^+^PCR^−^ and IFA^−^PCR^−^ groups showed that the Ct value was 40, indicating that HTNV RNA was not detectable in tissues." Was the Ct value 40, or not detectable? If HTNV RNA was not detectable, then the Ct value wouldn't be 40, more likely there would be no Ct value to mention. Please clarify here and on Figure 2.

**R**: Due to your comment, we re-wrote the sentence as shown below [Line 123-124].

**Line 123-124:**

Both IFA^+^PCR^−^ and IFA^−^PCR^−^ groups showed that HTNV RNA was undetectable in all tissues.

**4.** Results, lines 149-150: "The nucleotide similarity of the HTNV L, M, and S segments from Jeju Island compared to those of HTNV 76118 showed 82.2–83.1%, 87.2–88.8%, and 84.8–85.4% homology, respectively" Please check on the use of the terms "similarity" and "homology" in this sentence - would "identity" be more appropriate for nucleotide comparisons?

**R**: Based on the comment, we re-wrote the sentence as shown below [Line 151-152].

**Line 151-152:**

The nucleotide identity of the HTNV L, M, and S segments from Jeju Island showed 82.2–83.1%, 87.2–88.8%, and 84.8–85.4%, respectively, compared to those of HTNV 76-118 **(S2-S4 Tables)**.

**5.** Discussion, lines 263-264: "Taken together, the distinct genotype of A. chejuensis-harbored HTNV is considered a single species of A. agrarius-borne HTNV" The use of genotype and species in this sentence seem at odds with one another - it's not clear that "species" is the right use of the word in this context. On a similar note, please see the comment from a reviewer below about the use of the term "strain" - consider whether "sequence" or some other term might be more technically precise.

**R**: Thank you for your point. We re-wrote the sentence as shown below [Line 275-276].

**Line 275-276:**

Taken together, *A. chejuensis*-harbored HTNV is considered *Hantaan orthohantavirus* species, including *A. agrarius*-borne HTNV and *A. peninsulae*-borne SOOV (closely related to AMRV).

**6.** Discussion, lines 274-275: So, from the 18 clinical HFRS cases on Jeju Island over the past decade, there were no sequences available for comparison?

**R**: The etiological agent of HFRS patients on Jeju Island has not yet been investigated. Viral genomic sequences of the patients remain unavailable. Previous HFRS patients occured in Jeju Island were diagnosed by serologic test and clinical sysmptom [Lim et al., 2012. Case of Hemorrhagic Fever with Renal Syndrome in Jeju Island. Infection & Chemotherapy].

**7.** Discussion, lines 305-308: "The phylogenetic pattern of A. chejuensis-borne HTNV may be a configuration compatible with genetic reassortment. However, recombination and reassortment were considered insignificant by Recombination Detection Program 4 and Graph-incompatibility-based Reassortment Finder, respectively (data not shown)." I think it would be useful to include these tools in the Methods, particularly for the "reassortment finder" program, since it gave a result that is not obvious from the phylogenetic tree.
**R**: Based on your suggestion, we add the sentences as shown below [Line 245-248 and Line 404-416].

**Line 245-248:**

**Genetic reassortment analysis of HTNV**

Recombination Detection Program 4 (RDP4) and Graph incompatibility based Reassortment Finder (GiRaF) analyses were performed to evaluate the possibility of genetic reassortment events. The genetic exchange event was undetectable among HTNV genomic sequences in this study.

**Line 404-416:**

**Genetic reassortment analysis**

Alignment analysis of the concatenated HTNV L, M, and S segment ORF regions were performed using RDP, GENECONV, MAXCHI, CHIMAERA, 3SEQ, BOOTSCAN, and SISCAN in the RDP4 software (1). A p-value of recombination and reassortment events under 0.05 was considered statistically significant. Genetic events were likely to indicate the occurrence of a genetic exchange when P-value was under 0.05, and the RDP recombination consensus score (RDPRCS) was between 0.4 and 0.6. All parameters of RDP4 were left at the default.

The GiRaF was performed to confirm reassortment events (2). Nucleotide alignments of the tripartite segments were used as an input source for MrBayes (3). The best nucleotide substitution models were determined by using MEGA 7. As input for the software, 1,000 unrooted candidate trees were estimated using the GTR+G+I substitution model, the burn-in 50,000 iterations (25%), and sampling every 200 iterations. These trees were used to simulate the phylogenetic uncertainty for segments; the parameters of the GiRaF were default settings. The process was repeated ten times, with ten independent MrBayes based tree data per segment.

**Reviewer's Responses to Questions**
**8.** L 260: Species Demarcation criterion stated here seems to be outdated. Please check for more recent criterion in ICTV using concatenated amino acid sequences of N and Gn/Gc proteins of hantaviruses to demarcate the species. I agree novel sequences from Cheju islands were a novel "genotype" of Hantaan virus. But you should show results with new criteria.
**R:** Thank you for your point. Based on your suggestion, we checked the species demarcation criterion of orthohantavirus on the ICTV website [ICTV ICTV Ninth Report; Taxonomy Release. 2009. Available online: https://talk.ictvonline.org/ictvreports/ictv_9th_report/negative-sense-rna-viruses-2011/w/negrna_viruses/205/bunyaviridae (accessed on 29 Mar 2021)]. The ICTV 9^th^ report is an available latest chapter for the detection of species demarcation criterion of *Bunyaviridae*. The report reflects the recently current taxonomic information as shown below [Species demarcation criteria in the genus]. The species demarcation criterion of *Bunyaviridae* has not been included in the ICTV 10^th^ report.

**[Species demarcation criteria in the genus]**

Species are usually found in unique ecological niches, i.e. in different primary rodent/insectivore reservoir species. Species exhibit at least 7% difference in aa identity on comparison of the complete glycoprotein precursor and nucleocapsid protein sequences (there are some exceptions presumably caused by historically recent host-switching events). Species show at least four-fold difference in two-way cross neutralization tests. Species do not naturally form reassortants with other species.

**9.** Were correct statistical analysis used to support conclusions?
Why did you ignore Niviventer borne-Hantaan viruses in this manuscript? I can see the relationship of novel genotype and Amur viruses. In phylogenetic analyses, please include Niviveter borne-viruses. You discuss about reassortment events. For this purpose, More Chinese Hantaan viruses should be included in phylogeny.
**R**: Thank you for a good comment. Our study has become solid due to your opinion. Dàbiéshān virus (DBSV), harbored by Chinese white-bellied rat (*Niviventer confucianus*), belongs to the species of *Dabieshan orthohantavirus* [Abudurexiti et al., 2019. Taxonomy of the order *Bunyavirales*: update 2019. Archives of Virology]. Based on your suggestion, we added more genomic sequences of orthohantaviruses from China and conducted a phylogenetic analysis. We added and re-wrote the sentence as shown below [Line 163-164, Fig 3, and Line 315-317].

**Line 163-164:**

The S segment of HTNV from Jeju Island formed an independent genetic lineage with other HTNV, SOOV, and Amur virus (AMRV).

**Fig 3:**

**Fig 3. Phylogeographic analysis of the L, M, and S segments of Hantaan virus (HTNV) collected from rodents in Jeju Island, Republic of Korea (ROK).** Whole-genome sequences of HTNV from *A. chejuensis* of Jeju Island were obtained by multiplex polymerase chain reaction-based next-generation sequencing during 2018–2020. The phylogenies were generated by maximum likelihood methods (MEGA 7.0) with bootstrap 1,000 iterations based on the HTNV (A) L (1–6,533 nt), (B) M (1–3,618 nt), and (C) S (1–1,696 nt) segments, respectively. The branch lengths were proportional to the number of nucleotide substitutions, while vertical distances were used for clarity. The numbers at each node are bootstrap probabilities determined for 1,000 iterations. HTNV groups are color-coded according to geographic sites (red, mainland ROK; blue, Jeju Island of ROK; green, China). The brown color indicates SOOV and AMRV strains from East Asia (China, Russia, and ROK). The following hantaviral sequences were included in the analysis: HTNV Aa03-387 (L segment, KT934958; M segment, KT934992; S segment, KT935026), Aa04-722 (L segment, KU2071740; M segment, KU207182; S segment, KU207190), Aa05-190 (L segment, KT934959; M segment, KT934993; S segment, KT935027), Aa05-331 (L segment, KT934962; M segment, KT934996; S segment, KT935030), Aa09-410 (L segment, KU207177; M segment, KU207185; S segment, KU207193), Aa09-948 (L segment, KT934966; M segment, KT935000; S segment, KT935034), Aa14-172 (L segment, KT934974; M segment, KT935008; S segment, KT935042), Aa15-82 (L segment, MT012572; M segment, MT012560; S segment, MT012548), Aa15-84 (L segment, MT012573; M segment, MT012561; S segment, MT012549), Aa17-337 (L segment, MT012574; M segment, MT012562; S segment, MT012550), Aa17-353 (L segment, MT012575; M segment, MT012563; S segment, MT012551), Aa17-367 (L segment, MT012576; M segment, MT012564; S segment, MT012552), Aa17-421 (L segment, MT012577; M segment, MT012565; S segment, MT012553), Aa17-422 (L segment, MT012578; M segment, MT012566; S segment, MT012554), Aa18-164 (L segment, MT012579; M segment, MT012567; S segment, MT012555), Aa18-179 (L segment, MT012580; M segment, MT012568; S segment, MT012556), Aa18-185 (L segment, MT012581; M segment, MT012569; S segment, MT012557), Ac19-6 (L segment, MW219756; M segment, MW219762; S segment, MW219768), Ac20-5 (L segment, MW219757; M segment, MW219763; S segment, MW219769), Ac20-6 (L segment, MW219758; M segment, MW219764; S segment, MW219770), Ac20-30 (L segment, MW219759; M segment, MW219765; S segment, MW219771), Ac20-31 (L segment, MW219760; M segment, MW219766; S segment, MW219772), Ac20-32 (L segment, MW219761; M segment, MW219767; S segment, MW219773), 76-118 (L segment, NC_005222; M segment, M14627; S segment, M14626), HV004 (L segment, JQ083393; M segment, JQ083394; S segment, JQ093395), A9 (L segment, AF293665; M segment, AF035831; S segment, AF329390), Q32 (L segment, DQ371906; M segment, DQ371905; S segment, AB027097), 84FLi (L segment, AF336826; M segment, AF345636; S segment, AF366568), Z10 (L segment,NC_006435; M segment, NC_006437; S segment, AF366568), TJJ16 (L segment, KU215675; M segment, EU074672; S segment, AY839871), SOOV SC-1 (L segment, DQ056292; M segment, AY675353; S segment, AY675349), SC-2 (L segment, AY675354; M segment, DQ056293; S segment, AY675350), SC-3 (M segment, DQ056294; S segment, AY675351), SC-4 (M segment, DQ056295; S segment, AY675352), AMRV strains ApJLC2011-99 (L segment, JX473002; M segment, JX473003; S segment, JX473004), JilinAP06 (M segment, EF371454; S segment, EF121324), AP1371 (S segment, AF427324), AP61 (S segment, AB071183), DBSV Nc167 (L segment, DQ989237; M segment, AB027115; S segment, AB027523), and SEOV 80-39 (L segment, NC_005238; M segment, NC_005237; S segment, NC_005236). Abbreviations: SOOV, Soochong virus; AMRV, Amur virus; DBSV, Dàbiéshān virus; SEOV, Seoul virus.

**Line 316-318:**

The M segment formed a clade with HTNV strains in the ROK. The S segments showed the independent geographic and distinct cluster with *Hantaan orthohantavirus*, including HTNV, SOOV, and AMRV.

**10.** It has been reported that mice derived from Cheju island were used to isolate prototype Hantaan virus strain 76-118 virus by Dr. H. W. Lee (Hantavirus Hunting).  At that time, they explained that this island was HFRS patient-free, so that they used apodemus mice derived from Jeju island. But you clearly showed existence of Hantaan virus in apodemus mice in this island. You have to discuss about following for public health problem?
**R**: Jeju Island has been an endemic HFRS region since the first clinical case was officially identified in 2005. A total of 18 clinical cases have been reported in the recent decade on Jeju Island [Korea Disease Control and Prevention Agency (KDCA). Infectious disease portal. Available at: [http://www.cdc.go.kr/npt/biz/npp/ist/bass/bassDissStatsMain.do. Accessed 10 September 2020](http://www.cdc.go.kr/npt/biz/npp/ist/bass/bassDissStatsMain.do.%20Accessed%2010%20September%202020).]. Our findings showed that *A. chejuensis*-borne HTNV is genetically linked to CUH15-126 which is the only hantaviral sequence collected from the patient with HFRS in southern ROK (Jeollanam Province). These results arise awareness that *A. chejugensis*-borne HTNV may be a potential etiological agent in clinical HFRS cases in southern ROK. However, some questions should be more investigated: 1) the geographical distribution and prevalence of *A. chejuensis*-borne HTNV and their reservoir host populations in the central and southern areas of ROK; 2) whether HTNV harbored by *A. chejuensis* has the pathogenicity in humans; 3) the transmission and virulence mechanism of *A. chejuensis-*borne HTNV by isolating the virus. In this study, we described the public health problem of *A. chejuensis*-borne HTNV in the Discussion [Line279-308].

**Line 279-308:**

Rodent-borne orthohantaviruses pose a critical public health threat worldwide. In the ROK, approximately 400 HFRS cases are reported annually, with 33 mortality cases over a decade [7]. Approximately 70% HFRS cases are caused by *A. agrarius*-borne HTNV and the remaining by SEOV carried by *R. norvegicus* or other unidentified agents. Jeju Island includes urban and rural regions with a population of approximately 700,000. It is a popular tourism destination for international tourists to be listed as a Biosphere Reserve (2002), World Natural Heritage Site (2007), and Global Geopark (2010). Overall, visitation to the site has been sustainably increased by an influx of international tourists due to global reputations [38]. The island has been an endemic HFRS region since the first clinical case was officially identified in 2005. After five HFRS cases were recorded from 2001 to 2009, 18 clinical cases have been reported in the recent decade on Jeju Island [7]. Despite the distribution of the rodent reservoir population, HTNV and SEOV have not yet been detected on Jeju Island. In the present study, the genetically distinct HTNV was detected in *A. chejuensis* captured on Jeju Island. Phylogenetic analyses of orthohantaviruses delineated a clear molecular epidemiological link between the patients with HFRS and their reservoir hosts collected at the infection sites [39]. In particular, active surveillance with targeted rodent trapping in the sites suspected of orthohantavirus outbreaks associated with HFRS allowed for phylogenetic distinction within a relatively short distance (about 5 km) to track the precise infectious agents and potential exposed sites [14]. Our previous studies demonstrated a phylogenetic relationship of partial HTNV M segment genome between four US soldiers diagnosed with HFRS and HTNV-positive *A. agrarius* collected at the very likely infection sites [40]. The phylogenetic analysis showed that HTNV strains derived from *A. chejuensis* genetically linked CUH15-126, which is the only hantaviral sequence collected from the patients with HFRS in southern ROK (Jeollanam Province) [41]. These results indicate that *A. chejugensis*-borne HTNV may be a potential etiological agent in clinical HFRS cases in southern ROK.

The subdivision of DOBV genotypes (Dobrava, Kurkino, Saaremaa, and Sochi) was differentiated by the characteristics in their phylogeny, rodent reservoirs, and geographical distribution [42]. Despite their high genetic homology, different DOBV genotypes induce HFRS with varied severities. The case-fatality rate (CFR) of clinical cases due to the Dobrava genotype harbored by *A. flavicollis* was 10–12% [43-45]. The CFR of HFRS cases caused by the Sochi genotype harbored by *A. ponticus* was approximately 6% [46]. Furthermore, the CFR range for orthohantavirus outbreaks associated with the Kurkino genotype carried by *A. agrarius* was 0.3–0.9% in European Russia. *A. agrarius*-borne SAAV genotype infection appears to be subclinical [47, 48]. Thus, the transmission, pathogenicity, and disease risk assessment of HFRS by distinct *A. chejugensis*-borne HTNV in humans should be determined.

**11.** Is there any *Apodemus agrarius* genotype in this island?

**R**: The previous studies revealed that at least 22 species of rodents, insectivores, and bats, including reservoir hosts of hantavirus, inhabit on Jeju Island [Jo et al., 2012. Current status of terrestrial mammals on Jeju Island. Journal of Species Research]. However, the population of striped field mouse (*A. agrarius*) has not found in Jeju Island.

**12.** Is it possible that the Jeju-derived genotype is less pathogenic?
**R**: The pathogenicity of *A. chejuensis*-borne HTNV in humans has not been determined. Our further study aims to investigate the transmission and pathogenicity of *A. chejuensis*-borne HTNV in humans.

**13.** Since when has this virus been in this island? Host mouse was subspecies of *A. agrarius.* Rescently, it was considered independent species. Anyway, it was endemic only Cheju island. You have to clearly explain in explanation in Fig. 5
**R**: Thank you for a good comment. We may estimate that populations of *A. chejuensis* were infected with ancestral HTNV prior to the geological isolation between Korean peninsula and Jeju Island. Jeju Island of ROK was formed by a series of volcanic activities about 1.2 million years ago [Won et al., 1986. Paleomagnetism and radiometric age of trachytes in Jeju Island, Korea. Economic and Environmental Geology. Economic and Environmental Geology]. The island was connected to the Korean peninsula, mainland of ROK, during the Pleistocene and separated about 10,000 years ago. Previous studies may suggest that ancestral population of *A. chejuensis* on Jeju Island may have originated from the Korean Peninsula or eastern China [Oh et al., 2013. Migration route estimation of the Jeju striped field mouse *Apodemus agrarius chejuensis* (Rodentia, Muridae). Mitochondrial DNA]. A population of *A. agrarius* that are genetically and morphologically clustered with *A. chejuensis* has been reported in Wan Island (80km North from Jeju Island) located on the southern coast of ROK [Koh et al., 2000. The genetic relationships of two subspecies of striped field mice, *Apodemus agrarius coreae* and *Apodemus agrarius chejuensis*. Heredity]. Han et al. estimated the population of *A. agarius* and *A. chejuensis* was diverged about 1.2 million years ago, whereas Koh et al. suggested that these two species subdivided at 7,000–500,000 years ago (Pleistocene period), although both referred to the last ice age as a crucial period [Han et al., 1996. Variation of the mitochondrial DNA and the nuclear ribosomal DNA in the striped field mouse *Apodemus agrarius* on the mainland and offshore islands of South Korea. Mammal Study]. However, continued surveillance and large-scale investigations should be conducted for better understanding the evolutionary diversification and geographic distribution of orthohantavirus and their natural reservoir host in ROK.

We re-wrote and add the sentences as shown below [Line 259-264].

**Line 259-264:**

However, a population of *A. agrarius* that are genetically and morphologically clustered with *A. chejuensis* has been reported in Wan Island (80km North from Jeju Island) located on the southern coast of ROK [30]. Jeju Island was formed by a series of volcanic activities about 1.2 million years ago [31-34]. The island was connected to the Korean peninsula during the Pleistocene and separated about 10,000 years ago. The ancestral population of *A. chejuensis* on Jeju Island may have originated from the Korean peninsula or eastern China [35].

**14.** Reviewer #2: L 34: The sentence should better to be written as “The prevalence of anti-HTNV antibodies among rodents was 14.1%.”

**R**: Based on your suggestion, we re-wrote the sentence as shown below [Line 32-33].

**Line 32-33:**

The prevalence of anti-HTNV antibodies among rodents was 14.1%.

**15.** L 36: The sentence should better to be written as “The detected HTNV genomic sequences were phylogenetically related to a viral sequence derived from HFRS patients in southern ROK.”
**R**: Due to your comment, we re-wrote the sentence as shown below [Line 37-38].

**Line 37-38:**

The detected HTNV genomic sequences were phylogenetically related to a viral sequence derived from HFRS patient in southern ROK.

**16.** L 60: include the word “genome” before the word segments.
**R**: Based on the comment, we re-wrote the sentence as shown below [Line 60-61].

**Line 60-61:**

*Orthohantavirus* (Family *Hantaviridae*, Order *Bunyavirales*) is an enveloped negative-sense single-stranded RNA virus containing large (L), medium (M), and small (S) genome segments [1].

**17.** L 67: include “of infected rodents” after the word feces.
**R**: Thank you for your point. Based on your suggestion, we re-wrote the sentence as shown below [Line 67-68].

**Line 67-68:**

Orthohantaviruses are transmitted to humans by inhaling aerosolized infectious particles derived from saliva, urine, and feces of infected rodents.

**18.** Figure 1: Is there any special reason to capture rodents only from Jeju-si part of the island?
**R:** We have a limitation of trapping schedules and field situations (transportation methods and number or traps). Our further study aims to collect small mammals in the endemic area, including Seogwipo-si.

**19.** Figure 2: Have you tried calculating the copy numbers of RNA in tissues?
**R**: We conducted RT-qPCR for HTNV S segment. Based on your suggestion, the viral copy number of HTNV genomes was calculated using a described formula of linear regression curve in our previous study [No et al., 2019. Comparison of targeted next-generation sequencing for whole-genome sequencing of *Hantaan orthohantavirus* in *Apodemus agrarius* lung tissues. Scientific reports]. Ac20-5 and Ac20-31 harbored the highest amount of HTNV RNA (Ct values 21.8–23.8) corresponding to 10^4^ to 10^5^ copies/μL in the evaluated rodents. Ac20-30 and Ac20-32 showed Ct values ranging from 25.9–27.7 (10^3^ to 10^4^ copies/μL of viral RNA) in the HTNV positive *A. chejuensis* lung tissue. The Ct values of the rodents (Ac19-6 and Ac20-6) were 30.0–31.5, indicating that the samples contained 10^2^ to 10^3^ copies/μL of viral RNA.

**20.** Figure 4: The sequences for the phylogenetic analysis have been selected only from northern-most and southern-most regions of ROK. Is there a special reason not to select any sequence originated from central or other parts of the country?

**R**: Although HFRS cases have been reported in the central and southern Provinces of ROK, the studies about epidemiological surveillance of orthohantaviruses in human and rodent populations circulating in the central and southern areas of ROK were limited [Lee et al., 2013. Epidemiology of hemorrhagic fever with renal syndrome in Korea, 2001-2010. J Korean Med Sci]. The prevalence of HTNV was revealed from *A. agrarius* collected in the central and southern areas, but the available viral sequences have limitations [Lim et al., 2012. Seroprevalence of hantaviruses in small wild mammals trapped in South Korea from 2005 to 2010. J Vector Ecol].

**21.** Table 3: 
1) Add “from Jeju island” at the end of the table title.

**R**: Based on the comment, we re-wrote the sentence as shown below [Line 145].

**Line 145:**

Next-generation sequencing coverages of Hantaan virus (HTNV) from rodents collected in Jeju Island, 2018-2020.

2) The term “strain” refers to the isolated and established virus. These are basically the sequences detected from rodent tissues. Therefore, it is preferred to use another term instead of “strain” in all the places (especially in abstract) stating it in the manuscript. 
**R**: Thank you for your point. Based on your suggestion, we corrected and re-wrote the sentence as shown below [Line 33-35, Line 137-139, and Line 161-162].

**Line 33-35:**

The amplicon-based next-generation sequencing provided nearly full-length tripartite genomic sequences of six HTNV harbored by *A. chejuensis*.

**Line 137-139:**

The uncovered sequences of HTNV tripartite RNA were determined from HTNV Ac19-6, Ac20-5, Ac20-6, Ac20-30, Ac20-31, and Ac20-32, using conventional RT-PCR.

**Line 161-162:**

The L segment of HTNV from Jeju Island formed an independent geographic and distinct cluster with all other viral genomes collected from mainland ROK and China.

**22.** L 319: Is there an explanation for the low sample number even though the trapping was conducted for 2 years using relatively large number of traps? 
**R**: In total, 600 traps were set up for small mammals trapping on Jeju Island during 2018-2020. We collected a total of 70 rodents and shrews. The trapping rate was 11.7% (70/600) on Jeju Island. The rate is similar or a little low to our previous studies [Lee et al., 2020. Phylogeographic diversity and hybrid zone of *Hantaan orthohantavirus* collected in Gangwon Province, Republic of Korea. PLOS Neglected Tropical Diseases].

**23.** L 348: Why low dilutions were used for IFA assay? The background reactions can be quite higher at such a low dilution level.
**R**: Thank you for a good comment. We conducted the IFA test using the low dilution level with PBS in initial experiments for all trapping rodents and shrews. We re-conducted the IFA assay to confirm IFA titers using the 2-fold serial dilutions. IFA titers were described in Table 3 [Line 145].

**Line 145:**

Table 3. Next-generation sequencing coverages of Hantaan virus (HTNV) from rodents collected in Jeju island, 2018-2020.

| **Site** | **Sample** | **IFA for anti-HTNV IgG** | **Origin** | **Ct value** | **HTNV genomes, % coverage** | | |
| --- | --- | --- | --- | --- | --- | --- | --- |
|  |  |  |  |  | **L segment** | **M segment** | **S segment** |
| Ora-dong,  Jeju-si | Ac19-6 | 1:1,024^a^ | Lung | 30.0 | 81 | 96.6 | 98.1 |
|  | Ac20-5 | 1:32^b^ | Lung | 23.8 | 58.6 | 99.2 | 98.4 |
|  | Ac20-6 | 1:16^b^ | Lung | 31.5 | 41.4 | 97 | 98.4 |
| Bongseong-ri,  Jeju-si | Ac20-30 | 1:256^a^ | Lung | 25.9 | 93.4 | 99.1 | 98.4 |
|  | Ac20-31 | 1:256^a^ | Lung | 21.8 | 93 | 99 | 98.4 |
|  | Ac20-32 | 1:8^b^ | Lung | 27.7 | 91 | 99.1 | 98.4 |

Ac, *Apodemus chejuensis*; IFA, indirect immunofluorescence antibody test; IgG, immunoglobulin G; C_t_, cycle threshold; L, large; M, medium; S, small.

**^a^**; IFA test was performed from sera, **^b^**; IFA test was performed from heart fluids.

Genome coverage was calculated by obtaining consensus sequences matching the prototype HTNV 76-118.

The complete genome sequence lengths of HTNV are the L (6,533 nt), M (3,616 nt), and S segments (1,696 nt), respectively.

**24.** L 350/351: Why only a single secondary antibody was used to screen three species of rodent serum samples? Is there a cross reactivity to anti-mouse IgG in shrew and T. triton sera? In our experience the said secondary antibody does not react with shrew sera.

**R**: Thank you for your point. We agree to your opinion. We conducted IFA test using mouse and rat secondary antibody for shrews (*Crocidura shantungensis*). We re-wrote the sentence as shown below [Line 363-365].

**Line 363-365:**

The slides were treated with fluorescein isothiocyanate-conjugated anti-mouse (for *A. chejuensis* and *T. triton*) or mouse/rat (for *C. shantungensis*) IgG (ICN Pharmaceuticals; Laval, Quebec, Canada).

**25.** L 361: RT-qPCR primers were targeted at M-segment of HTNV. Is there any reason to choose M-segment over S-segment which has more copies in the virus for the quantification?

**R**: Thank you for a good comment. We agree to your opinion. Due to your comment, we conducted RT-qPCR for HTNV S segment. We re-wrote the sentence and figure as shown below [Line 125-128 and Line 130-132].

**Line 125-128:**

The lung tissues harbored the highest amount of HTNV RNA (Ct values 21.8–31.5) in all evaluated rodents except Ac20-32. The brain tissues showed the presence of HTNV RNA in all rodents except Ac20-6. The rodents (Ac20-5, Ac20-31, and Ac20-32) contained the HTNV RNA in reproductive organs.

**Line 130-132:**

**Fig 2. Measurement of HTNV RNA loads in different tissues of *Apodemus chejuensis*, Jeju Island during 2018–2020.** Ct values were determined for HTNV RNA of the S segment in brain, lung, liver, kidney, spleen, reproductive organ, and heart tissues obtained from six rodents positive for HTNV IgG and HTNV RNA. Ct, cycle threshold; S, small; HTNV, Hantaan virus; IgG, immunoglobulin G.

Reviewer #1: The study entitled “A novel genotype of Apodemus chejuensis-borne Hantaan Orthohantavirus as a potential etiologic agent of Hemorrhagic Fever with Renal Syndrome in Republic of Korea” appears to be a fascinating approach to identify the novel genotype as a pivotal etiologic agent of HFRS. 
       However, there are certain minor areas in which the author needs to clarify in order to refine the work for better representation.
**26.** Line no. 25 – Repetition of the word “pulmonary”. Hantavirus cardiopulmonary syndrome (HCP)

**R**: Thank you for your point. Based on your suggestion, the term was corrected as shown below [Line 24-25].

**Line 24-25:**

Orthohantaviruses, causing hemorrhagic fever with renal syndrome (HFRS) and hantavirus cardiopulmonary syndrome, pose a significant public health threat worldwide.

**27.** Line 34 - The expression of sentence is not very clear. What does the author mean to say, should clearly explain?

**R**: We re-wrote the sentence as shown below [Line 35-36].

**Line 35-36:**

Phylogenetic and tanglegram analyses were conducted for inferring evolutionary relationships between orthohantaviruses with their reservoir hosts.

**28.** Line 87 - HFRS-Borne or Rodent-borne? HTNV is the causative agent of HFRS. 
•       What does the author mean to say by the phrase clinical HFRS-borne HTNV strain?

**R**: Thank you for a good comment. we re-wrote the sentence as shown below [Line 87-89].

**Line 87-89:**

The phylogenetic association of *A. chejuensis*-borne HTNV with viral sequences from HFRS patient suggests that *A. chejuensis*-borne HTNV may be a potential etiological agent of HFRS on Jeju Island, ROK.

**29.** Line 95 – 64 should be replaced with sixty-four.

**R**: Based on the comment, we re-wrote the sentence as shown below [Line 96-98].

**Line 96-98:**

The trapped small mammals consisted of 91.4% (64/70) *A. chejuensis*, 5.7% (4/70) *C. shantungensis*, and 2.9% (2/70) *Tscherskia triton* (Table 1).

**30.** Line 108 - The author has mentioned about the collection of shrews and rodents both for the experiment. But the data related to shrews are missing.
•       Was there any seropositive case for shrews as well?

No. The anti-hantavirus antibody was not detected in captured *C. shantungensis* and *Tscherskia trition* in this study. We re-wrote the sentence as shown below [Line 117-118].

**Line 117-118:**

The antibodies against orthohantaviruses were not detected in *C. shantungensis* and *T. triton*.

**31.** Line 124 – What was the estimated cut-off value for the RT-qPCR. Author did not mention about that.
**R**: Due to your comment, we re-wrote the sentence as shown below [Line 123-124].

**Line 123-124:**

Both IFA^+^PCR^−^ and IFA^−^PCR^−^ groups showed that HTNV RNA was not detectable in all tissues.

Reviewer #3:
**32.** First concerns:
Though not necessarily a “major” concern, this reviewer feels it is important to mention that taxonomically, Apodemus chejuensis is most likely the sub-species of Apodemus agrarius chejuensis (see <https://www.ncbi.nlm.nih.gov/Taxonomy/Browser/wwwtax.cgi?id=754351> and <https://www.iucnredlist.org/species/1888/115057408>). However, if A. chejuensis is genetically distinct from mainland A. agrarius, the authors could also claim as a new species? (see lines 97-98 S1 Fig).
There may be some push-back from the evolutionary mammalogist community if this is not mentioned. This can be solved in the introduction of A. chejuensis (line 86). Example: 
ln 86 “. . . orhtohantavirus, harbored by the subspecies A. agrarius chejuensis (hereafter, A. chejuensis) captured on Jeju Island.”
**R**: Thank you for your point. Based on your suggestion, we re-wrote the title as shown below [Line 86-87].

**Line 86-87:**

In this study, we report the nearly full-length genome sequences and characterize a genetically distinct orthohantavirus, harbored by the subspecies *A. agrarius chejuensis* (*A. chejuensis*) captured on Jeju Island.

**33.** Check throughout to remain consistent with “orthohantavirus” replacing “hantavirus”. (e.g. lns 66, 69,79,80,86, etc.; it’s hard for this reviewer to get used to also!)
**R**: Based on the comment, the term was corrected to remain consistent [Line 26, 28, 43, 53, 57, 67, 68, 70, 73, 80, 81, 87, 290, 292, 305, 335, and 339].

Minor concerns:
**34.** Title ln2: might consider adding full subspecies epithet.
**R**: Due to your suggestion, we re-wrote the title as shown below [Line 2-3].

**Line 2-3:**

A novel genotype of *Hantaan Orthohantavirus* harbored by *Apodemus agrarius chejuensis* as a potential etiologic agent of Hemorrhagic Fever with Renal Syndrome in Republic of Korea

**35.** ln27: “orthohantaviruses”
**R**: Based on your suggestion, the term was corrected as shown below [Line 26-28].

**Line 26-28:**

This study aimed to investigate the prevalence of HFRS-associated orthohantaviruses and identify the etiological agent of hantavirus outbreaks in southern Republic of Korea (ROK).

**36.** ln32: same type of introduction of subspecies (if decided to go that route).; not sure “respectively is needed?
**R**: No. we removed the term in the sentence [Line 30-32].

**Line 30-32:**

We detected the Hantaan virus (HTNV)-specific antibodies and RNA using an indirect immunofluorescence assay test and reverse transcription-polymerase chain reaction on *Apodemus agrarius chejuensis* (*A. chejuensis*).

**37.** ln35: show (n=x/y) for percentages?
**R**: Thank you for your point. We re-wrote the sentence as shown below [Line 33].

**Line 33:**

A total of six seropositive mice harbored HTNV RNA.

**38.** ln55: “significant” might consider different word; could imply the need for statistical evidence.
**R**: Thank you for a good comment. Based on your suggestion, the term was corrected as shown below [Line 56-57].

**Line 56-57:**

This study provides important insights into the male-predominance prevalence, genetic diversity, and molecular evolutionary dynamics of hantaviruses circulating in the ROK.

**39.** ln64: taxonomic orders do not need italics; change “Soricomorpha” to “Eulipotyphla”
**R**: Based on the comment, the term was corrected as shown below [Line 64-65].

**Line 64-65:**

Natural reservoirs of orthohantaviruses belong to various small mammals, including rodents (Rodentia), bats (Chiroptera), and insectivores (Soricomorpha) [3-5].

**40.** ln81: change “In” to “On”?
**R**: Based on your suggestion, the term was corrected as shown below [Line 82-83].

**Line 82-83:**

On Jeju Island, an administrative island in southern ROK, approximately 18 HFRS cases have been reported from 2011 to 2019 [7].

**41.** ln94-95: suggested re-write: “The trapped small mammals consisted of 91.4% (64/70) A. chejuensis and 2.8% (2/70) T. triton rodents and 2 C. shantugensis shrews.”
**R**: Due to your comment, we re-wrote the sentence as shown below [Line 96-98].

**Line 96-98:**

The trapped small mammals consisted of 91.4% (64/70) *A. chejuensis*, 5.7% (4/70) *C. shantungensis*, and 2.9% (2/70) *Tscherskia triton* (Table 1).

**42.** ln102-104: Fig 1 and caption, it might be helpful to place a site number in the text and on the figure.
**R**: Thank you for your point. Based on your suggestion, we modified the figure as shown below [Line102-107].

**Line 102-107:**

**Fig 1. Geography of trapping sites of Hantaan virus (HTNV) collected on Jeju Island, the Republic of Korea.** A geographic map shows different trapping locations where small mammals were captured on Jeju Island from 2018 to 2020. The red circles indicate the HTNV RNA positive areas: a, Ora-dong; b, Bongseong-ri in Jeju-si, respectively. The black circle represents the regions where no HTNV RNA was identified: c, Ara-dong; d, Aewol-eup; e, Haengwon-ri; f, Youngsu-ri. The Quantum Geographical Information System (QGIS) 3.10 for Mac was used to create the map.

**43.** ln106: consider “Trapping results of small mammals collected at sites on Jeju Island in the Republic of Korea, 2018-2020.”
**R**: Based on the comment, we re-wrote the sentence as shown below [Line 108-109].

**Line 108-109:**

Table 1. Trapping results of small mammals collected at sites on Jeju Island in the Republic of Korea, 2018–2020.

**44.** ln107: bottom of table, “no collection” descriptor is likely not necessary 
**R**: Based on your suggestion, we removed the descriptor in the sentence.

**45.** lns114-115: sentence is a little confusing. It suggests to the reader that the tests (antibodies vs RNA) were exclusive to host and not used for each.
**R**: Thank you for your point. We re-wrote the sentence as shown below [Line 117-118].

**Line 117-118:**

The antibodies against orthohantaviruses were not detected in *C. shantungensis* and *T. triton*.

**46.** ln135 & 640-641: S1 Table might consider reducing the large number values to 3-4 digits with the conversion metric in column header of the table (e.g. total reads, reads mapped, and depth of coverage).
**R**: Based on the comment, we re-wrote the sentence as shown below [Line 681-682].

**Line 681-682:**

**S1 Table. Summary of mapped reads and depth of coverages by Hantaan virus (HTNV) multiplex PCR-based next-generation sequencing.**

| **Sample** | **Total reads** | **Mapped reads /**  **Total reads (%)** | **L segment** | | **M segment** | | **S segment** | |
| --- | --- | --- | --- | --- | --- | --- | --- | --- |
|  |  |  | **Mapped**  **reads (%)** | **Depth of coverage** | **Mapped**  **reads (%)** | **Depth of coverage** | **Mapped**  **reads (%)** | **Depth of coverage** |
| Ac19-6 | 720,838 | 11.8 | 2,794 (0.4) | 64.6 | 35,966 (5.0) | 1,501.9 | 46,629 (6.5) | 4,151.5 |
| Ac20-5 | 2,287,084 | 56.3 | 21,516 (0.9) | 497.3 | 755,097 (33.0) | 31,532.0 | 511,299 (22.4) | 45,522.5 |
| Ac20-6 | 1,597,734 | 28.5 | 6,276 (0.4) | 145.1 | 27,269 (17.1) | 1,138.7 | 421,325 (26.4) | 37,511.8 |
| Ac20-30 | 1,141,262 | 44.6 | 30,952 (2.7) | 715.4 | 297,141 (26.0) | 12,408.3 | 180,933 (15.9) | 16,109.0 |
| Ac20-31 | 1,547,806 | 32.5 | 32,313 (2.1) | 746.9 | 359,727 (23.2) | 15,021.8 | 111,552 (7.2) | 9,931.8 |
| Ac20-32 | 2,615,908 | 30.5 | 53,036 (2.0) | 1,225.8 | 549,311 (21.0) | 22,938.6 | 195,606 (7.5) | 17,415.4 |
| **Average** | **1,651,772** | **34.0** | **24,481 (1.5)** | **565.9** | **337,419 (20.4)** | **16,607.9** | **244,557 (14.8)** | **21,773.7** |

Ac, *Apodemus chejuensis*; C_t_, cycle threshold; L, large; M, medium; S, small.

The depth of coverage was determined by the number of mapped reads (read length × number of reads matching the reference/reference genome size)

**47.** ln152-153: not sure the last sentence is needed. 
**R**: Thank you for your point. Based on your suggestion, we removed the sentence in the manuscript.

**48.** ln156: after “positions 644-648” does this refer to S3 Table? May want to include table reference.
**R**: Thank you for a comment. The positions 644-648 indicate the amino acid position of ‘WAASA’ on the M segment. ‘S3 Table’ showed the comparison of nucleotide and amino acid sequences among orthohantaviral M segments. Thus, we think the positions 644-648 is not associated with the S3 Table.

**49.** ln257: consider a new paragraph starting with the last “The . . .”
**R**: Thank you for your point. We modified the paragraph [Line 251-278].

**Line 251-278:**

Hantaviruses have specifically evolved along with one or a few closely related rodent species [4, 5, 24]. The genus *Apodemus* (family *Muridae*, order *Rodentia*) consists of at least 20 small rodent species and is mainly widespread in the Palearctic area [25]. Molecular data based on the rodent mtDNA cytochrome *b* gene and the interphotoreceptor retinoid binding protein gene in nuclear DNA revealed that *Apodemus spp*. diverged approximately 8–10 million years ago [26]. In Asia, *A. agrarius* was first identified as the primary rodent reservoir of HTNV [8]. *A. peninsulae* was also a rodent reservoir for SOOV (closely related to AMRV) distributed in ROK, China, and Russia [10, 27, 28]. The striped ﬁeld mouse (*A. agrarius coreae*), the most abundant rodent species, are widely distributed in the Korean peninsula, whereas *A. chejuensis* is mainly found on Jeju Island, the southernmost island of ROK [29]. However, a population of *A. agrarius* that are genetically and morphologically clustered with *A. chejuensis* has been reported in Wan Island (80km North from Jeju Island) located on the southern coast of ROK [30]. Jeju Island was formed by a series of volcanic activities about 1.2 million years ago [31-34]. The island was connected to the Korean peninsula during the Pleistocene and separated about 10,000 years ago. The ancestral population of *A. chejuensis* on Jeju Island may have originated from the Korean peninsula or eastern China [35]. Han et al. and Koh et al. estimated the population of *A. agrarius* and *A. chejuensis* diverged approximately 12 million years ago and 7,000–500,000 years ago (Pleistocene period), respectively, although both studies referred to the last ice age as a crucial period [30, 36]. *A. agrarius* and *A. chejuensis* have recently been suggested to be separate genera of *Apodemus spp*. based on mt genome analysis, morphological characteristics, and crossbreeding experiments [30, 36, 37]. The present study proposes that *A. chejuensis*-borne HTNV is a genetically distinct genotype due to the clear natural host species classification and co-divergent evolution of HTNV and *Apodemus spp*. after the geographic segregation between Jeju Island and the Korean peninsula. The species demarcation criterion of the International Committee for Taxonomy of Viruses (ICTV) is currently a 7% difference in amino acid sequences of GN/GC and N proteins. The differences in amino acid sequences of the RdRp, glycoproteins, and N proteins of HTNV strains harbored by *A. chejuensis* and *A. agrarius* did not exceed 5%. Taken together, the distinct *A. chejuensis*-harbored HTNV is considered *Hantaan orthohantavirus* species, including *A. agrarius*-borne HTNV and *A. peninsulae*-borne SOOV (closely related to AMRV). Continued surveillance and large-scale investigations should be conducted to understand the evolutionary diversification and geographic distribution of *orthohantavirus* and their natural reservoir hosts in ROK.

**50.** ln257-266: The authors make a very compelling argument, and this reviewer agrees with their conclusions.
ln286-289: Again, the authors have provided important information for the medical community as stated in their claims.
**R**: Appreciate your comment.

**51.** ln313: sentence is a little confusing; suggest changing to: “The seroprevalence of orthohantaviruses in rodents demonstrated a higher prevalence of infection in males than females.”
**R**: Based on the suggestion, we re-wrote the sentence as shown below [Line 325-326].

**Line 325-326:**

The seroprevalence of orthohantaviruses in rodents demonstrated a higher prevalence of infection in males than females [16, 53-57].
